# Supplementary material for: Structure and antigenicity of the divergent human astrovirus VA1 capsid spike
Source: PLoS Pathog. 2024 Feb 28;20(2):e1012028. doi: 10.1371/journal.ppat.1012028 (PMC10950212; doi:10.1371/journal.ppat.1012028)
Supplement: S1 Fig — Electron density maps (slate blue) are contoured at 1.0σ around the indicated amino acids. Regions were selected to highlight the electron density around amino acids that differ between the strains, for example at (A) amino acids 421–423, (B) amino acids 487–489, and (C) amino acids 572–574. (PDF) [file ppat.1012028.s001.pdf]

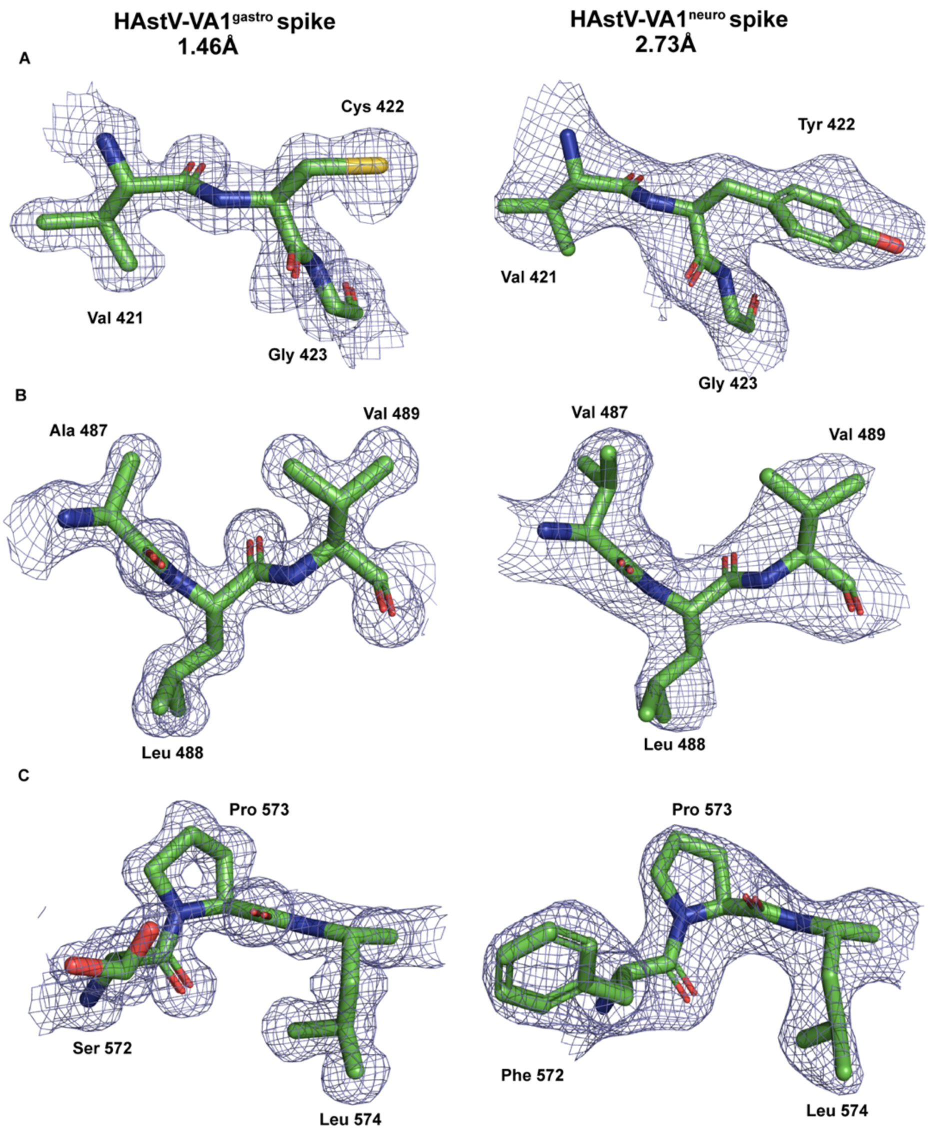

Supplemental Fig 1. Electron density maps of the HAstV-VA1^{gastro} spike and the HAstV-VA1^{neuro} spike. Electron density maps (slate blue) are contoured at 1.0σ around the indicated amino acids. Regions were selected to highlight the electron density around amino acids that differ between the strains, for example at (A) amino acids 421-423, (B) amino acids 487-489, and (C) amino acids 572-574.
