# Supplementary material for: Structure and antigenicity of the divergent human astrovirus VA1 capsid spike
Source: PLoS Pathog. 2024 Feb 28;20(2):e1012028. doi: 10.1371/journal.ppat.1012028 (PMC10950212; doi:10.1371/journal.ppat.1012028)
Supplement: Attachment — Submitted filename: Response_to_reviewers.docx [file ppat.1012028.s002.docx]

**Response to Reviewer Comments**

Reviewer comment

Response

We thank the reviewers for their thoughtful comments and suggestions. We believe that the changes improve the clarity and impact of the paper. All line numbers reported below are referring to those in the “Marked up manuscript” file.

**Reviewer #1:**

**0. The authors indeed show by BLI that a polyclonal rabbit antibody binds less well to the “neuro” strain than to the “gastro” strain. Yet, since in the Methods section they say that they used “hyperimmune rabbit serum to HAstV-VA1gastro virus”, this result was to be expected: the differences all map to the exposed spike surface, and so the serum will bind to the particles that were used to elicit it better than those that have multiple mutations at their surface.**

The reviewer is correct that it was somewhat expected that the hyperimmune serum would recognize the gastro strain better than the neuro strain, since the gastro strain was used to immunize the animals. However, the fact that we indeed found differences in the recognition of the two VA1 spikes is important to show experimentally, to support the idea that the amino acid changes in the neuro VA1 spike reduced the recognition of antibodies to the virus, and thus, they could have been selected in the immunocompromised host upon a prolonged replication.

**1. The abstract and the Author’s summary say the same thing. The latter could be used to place the context of the study, rather than repeating what is described in the abstract.**

We agree with the reviewer’s suggestions and edited the author summary to provide context to the study (lines 46-60).

**2- The conclusion of the study should be spelt out in the abstract, beyond saying that it provides new insight without stating which insights. (Yet this conclusion is not clearly stated, as the authors simply say that their study “provides insights into neurotropism and immune escape” without explaining what this insight is. This needs to be fixed, both in the abstract and at the end of the introduction (where they state it twice, in a redundant way).**

We agree with the reviewer’s suggestions and have edited the abstract (lines 28-29 and 32-33) and the end of the introduction (lines 153 and 164-166).

**3- The identification of the right boundaries for the spike domain should allow the authors to make a construct that diffracts better than the one for the “neuro” strain, for which the crystallographic data are sub-optimal.**

The resolution achieved for the “neuro” strain spike was sufficient for all the conclusions drawn in this manuscript. Evidence for the quality of the crystallographic data is discussed below.

**4- Since an important result of the study is the characterization of the way the VP86 precursor protein is characterized, with the boundaries of the mature VP33 and VP38 proteins, the authors should present side-to-side their results with those obtained earlier for the classical astroviruses (which have a VP90 precursor that leads to mature VP34 and VP27 proteins). A structural alignment of the corresponding proteins between the classical and the VO/VMA clade, highlighting the common secondary structures and maturation cleavage sites would be very useful.**

We completely agree and appreciate the insightful suggestion provided by the reviewer. In response, we have revised this figure (now Fig 4) and added panel D. We now show the cleavage sites in the classical HAstV capsid, which allows for comparison to the cleavage sites in the HAstV-VA1 capsid. We have also modified the manuscript at lines 315-316.

**Finally, here I pick some sentences of the manuscript that should be fixed: In the abstract (line 28) and Discussion (line 331) the authors write “suggesting that HAstV28 VA1 utilizes a different cell receptor.” I recommend writing “suggesting that it may utilize a different receptor”, to make clear that this is not known.**

We agree and have incorporated the recommended modifications by introducing the term "may" in both abstract (line 28) and in discussion (line 421).

**Lines 40-43: “The human astrovirus VA1 spike structure reveals major differences in size, shape, and structural features compared to the classical human astrovirus spike, suggesting that the divergent strains have evolved different mechanisms to attach to host cells”. This sentence needs rephrasing. What is meant by “major” here? The surface of spike is complete different. Pull our structure and show a alignment of those structure. Certainly not a different fold, as the secondary structure is the same. And does it necessarily mean that the attachment mechanism will be different? As an example that contradicts their assertion, the human alphacoronavirus N63 has a receptor binding domain in the spike protein that is unrelated to that of SRAS CoV-2, yet both use human ACE2 as receptor. So, I urge the authors to be more cautious in their assertions.**

We agree with the reviewer that the phrase “major differences” is scientifically vague. As such, we have replaced our use of “major differences” with “striking differences” at lines 51, 156, and 243, and we further describe the observed differences in size, shape, and biochemical surface at lines 244-252. We also added lines 254-258 to further justify our hypothesis of a different receptor: “Altogether, these size and structural differences result in a completely different biochemical surface on the HAstV-VA1 spike compared to the classical and MLB spikes, with no obvious three-dimensional patches of sequence homology that might indicate a conserved receptor-binding site.” We completely agree that the structure alone is insufficient evidence for the use of a different receptor, and the reviewer points to a perfect example of different virus structures binding to the same receptor. Thus, we are pleased to report new data from an *in vitro* infection competition assay (Fig 3 and lines 260-272) that further supports the hypothesis that classical and VA/HMO human astroviruses may utilize a different receptor.

**Line 120: “These structures provide insights into HAstV121 VA entry, evolution, maturation, tropism, and antigenicity.” As indicated above, please explain what these insights are, rather than repeating that there are new insights**

Following this sentence (line 152-153), the specific insights are described in the following lines 153-166, which now starts with “Specifically,”.

**Lines 127-129: “Mapping of variants within the spike domain of gastrointestinal and neuronal HAstV-VA1 strains yields insights into virus tropism and antigenicity, which are further supported by antigenic studies.” Same as above**

This line is now deleted and replaced at lines 164-166.

**129-132: “Overall, these studies provide valuable new insights that lay a foundation for the understanding of HAstV-VA biology and the development of vaccines and therapeutics against this divergent HAstV.”(Lines 377-381) described in details. Same as above.**

This statement (lines 166-168) is now edited for clarity.

**Lines 203-206: the loops and ying/yang motifs are not very apparent in the Figure. Please make an improved Figure, better highlighting the motifs mentioned in the text.(TOP VIEWS)**

We agree and have edited Fig 2 to incorporate top views for all of the spike structures.

Reviewer's Responses to Questions

**Part I - Summary**

**Please use this section to discuss strengths/weaknesses of study, novelty/significance, general execution and scholarship.**

**Reviewer #1: The manuscript by Gosh, et al., describes the structure of the VA1 astrovirus capsid spike using crystallography and sequence alignments. In immunocompromised patients, the virus can infect the nervous system. Therefore, the goal here was to compare those strains that cause limited gastrointestinal symptoms with those that cause neuropathology.**

**In brief, there are some interesting results presented here, but some major questions remain with regard to the quality of the neuro structure and impact of the results. Therefore, regrettably, I have to recommend ‘reject’.**

**Problems that need to be addressed.**

**1) One problem of concern was some of the data statistics shown in Table 1. The Rmerge statistics for the neuro strain are horrible. As written in the table, the overall Rmerge for the data is 35%, which is already very high, and the Rmerge for the highest resolution shell (2.7Å) is 210%. I frankly have never seen published numbers this bad. This not only clearly says that their resolution is not 2.7Å but, to me, casts doubt on their structure and its interpretation. Not surprisingly, the overall B factor for this structure is about 4 times that of the gastro strain. Solutions: A) the authors need to cite a realistic resolution that may impact how much it can be used in comparative analyses. B) example electron density needs to be shown so that the reader can evaluate the cited differences.**

There has been a shift over the past ~10 years in the understanding of how to use data quality indicators to choose a high-resolution cutoff to get the best model (see reviews “Assessing and maximizing data quality in macromolecular crystallography” by Karplus and Diederichs in 2015 and “The Resolution in X-ray Crystallography and Single-Particle Cryogenic Electron Microscopy” by Dubach and Guskov in 2020). While the traditional indicators were the Rmerge and I/σI, this approach is outdated. First, Rmerge is inherently flawed, as it is dependent on multiplicity, and its value increases with more measurements. We note that in our VA1 Spike^neuro^ structure, the multiplicity is 13.0 overall and 12.6 in the highest resolution shell, resulting in high Rmerge values. However, high multiplicity has been shown time and again to enhance measurable signal through decreasing noise, even with high Rmerge values, resulting in improved electron density maps and lower Rfree values of the final model (these studies are discussed in the aforementioned reviews).

Following modern practices, we utilized the Pearson’s Correlation Coefficient (CC_1/2_), as a quality indicator to determine the high-resolution cutoff. While there is not a single CC_1/2_ value used for resolution cutoff, generally somewhere between 0.2 - 0.5 is selected, along with evaluation of the I/σI. Thus, we actually feel conservative in selecting the 2.73 Å resolution cutoff, which has a CC_1/2_ value of 0.616 and I/σI of 1.5 in the highest-resolution shell. We added lines 591-593 to explain our use of CC_1/2_ and I/σI to select the resolution for this structure.

Nevertheless, we are happy to accept the reviewer’s suggestion and now include Fig S1 to illustrate the electron density maps from different regions of the spikes from HAstV-VA1^gastro^ and HAstV-VA1^neuro^. We purposely focused on amino acids that differ between the two structures to show that the electron density maps do support the modeling of those amino acid sidechains. We believe that the statistics in Table 1 and the Fig S1 electron density maps demonstrate the quality of both of the structures, allowing for the interpretations described in the manuscript.

**2) If using the region of 408-684 of gastro crystallized into such highly diffracting crystals, why was this not tried with the neuro? Clearly, there are issues with the neuro crystals. If this was attempted and failed, that should be noted.**

Please see above for our discussion on the quality of the HAstV-VA1^neuro^ spike crystal data and structure. While we did produce the indicated HAstV-VA1^neuro^ spike protein construct (amino acids 408-464) for ELISA and BLI studies, we did not attempt to crystallize it.

**Therein could lie a major difference between neuro and gastro. What if there is not a structural difference between neuro and gastro but a stability difference not observable in a crystal structure?**

Both neuro and gastro spike proteins were purified by size-exclusion chromatography and no aggregate / void volume peaks were observed (see Fig 1D). While not quantitative in measuring stability (i.e. via Tm measurements), the size-exclusion chromatography data support that the proteins are both stable as dimers in solution and not only the crystal.

**3) Gastro has six variable AA sites compared to other astroviruses whereas neuro has 27. These differences between neuro and gastro are scattered all over the spike. While the authors conclude that one or more of these differences are correlated with tissue tropism, there is not data to support this. This comes back to a nagging question I had with this study. Neuropathology is only seen in immunocompromised patients. I would posit that it is possible that both strains are able to infect the nervous system but is neutralized in normal patients before it can spread to that tissue. Therefore, are we really looking at fortuitous tropism differences or true differences in the capsids? Indeed, the antibody binding analyses suggest that the major differences between neuro and gastro are likely due to divergence due to immunotherapy in the immunocompromised neuro patients. This is a major concern to me since it speaks to impact of these studies. May contribute to propensity or astrovirus.**

We thank the reviewer for the comments and believe that we are making the same conclusions from the data – i.e. that the major differences between neuro and gastro spikes are likely due to escape from immunotherapy – and we acknowledge that our writing must not have clearly conveyed this. Also, while we did not conclude that differences are correlated with tissue tropism, we did originally introduce our experiments to say that we were investigating if sequence or structural changes were driving neurotropism (i.e. at line 335). However, we believe that we incorrectly used this word and have removed all uses of “tropism”, “neurotropism”, and “neurotropic” from the manuscript except for one instance in the abstract where we believe it is appropriately used (line 24). Instead, we use phrases like “influence its ability to infect the central nervous system” (line 445). We believe this phrase is appropriate because, while neuronal HAstV infections are rare, the majority of documented cases are due to HAstV-VA1, supporting that it may have an increased ability compared to classical and MLB HAstVs to spread beyond the intestinal tract in immunocompromised individuals. Thus, we frame our discussion to discuss possible reasons why, including differences in receptor usage and protease maturation.

**Reviewer #2:**

**The manuscript is well-written, with a good background, adequate methodological details, well-described results, an appropriate discussion, and well-designed figures.**

We thank the reviewer for the comprehensive summary and positive comments.

**The results are convincing, although their implications for why certain strains are associated with neurological diseases are tenuous.**

We agree that the structure alone was insufficient evidence for different receptor usage, which is one possible reason for why certain strains are associated with neurological diseases. We are pleased to report new data from an *in vitro* infection competition assay (Fig 3 and lines 260-272) that further supports the hypothesis that classical and VA/HMO human astroviruses use a different receptor. In the discussion section, we describe that both receptor usage and protease maturation may explain the higher rates of neurological disease by HAstV-VA1 strains.

**Other comments:**

**Abstract: lines: 29-33. These sentences, in present continuous tense (also lines 129-130), are a bit odd to read and non-informative. Authors should clearly state what they learned from these studies.**

We agree and have edited those lines for clarity (lines 29-31 and lines 164-166).

**Table 1: Overall Rmerge of ~35% and 210% for the highest resolution shell for the neuro data indicate poor quality and likely much lower resolution. The Rwork/Rfree values and their spread also are a bit problematic. Authors should include some explanation and comments if this affects the modeling and comparison with gastro structure.. .**

Please see the detailed response to reviewer #1 concerns with the Rmerge. We now include Fig S1 to illustrate the electron density maps from different regions of the spikes from HAstV-VA1^gastro^ and HAstV-VA1^neuro^. Regarding the Rwork/Rfree, we prioritized Ramachandran restraints and low RMSD for bond lengths and angles during model building and refinement to ensure a trustworthy structure, but this strategy can come at a slight cost to the Rwork/Rfree values. Finally, we note that after finishing the VA1^neuro^ spike structure, we then solved the VA1^gastro^ spike structure, and this higher-resolution structure resulted in an RMSD of only 0.78 Å and TM-score of 0.97 compared to the VA1^neuro^ spike structure, further supporting the validity of the VA1^neuro^ spike structure.

**Given high multiplicity, Rpim values for both the data sets should be included in the table.**

We agree and added Rpim values into Table 1.

**Lines 179, 366, 367, 456: gastrointestinal is pelt wrong.**

We thank the reviewer for pointing this out. We corrected the gastrointestinal spelling at lines 224, 469, 471, and 562.

**Part II – Major Issues: Key Experiments Required for Acceptance**

**Please use this section to detail the key new experiments or modifications of existing experiments that should be absolutely required to validate study conclusions.**

**Generally, there should be no more than 3 such required experiments or major modifications for a "Major Revision" recommendation. If more than 3 experiments are necessary to validate the study conclusions, then you are encouraged to recommend "Reject".**

**Reviewer #1: 1) There are issues with the structure of the neuro strain that need to be addressed.**

Please see our responses above.

**2) While probably beyond the scope of these studies, they need to demonstrate that there are truly differences in tissue tropism - probably via tissue culture analyses in the absence of an immune response. This speaks to the impact of these studies, which is a major concern**

We are pleased to report new data from an *in vitro* infection competition assay (Fig 3 and lines 260-272). While not directly demonstrating tissue tropism, these studies support the hypothesis that classical and VA/HMO human astroviruses may utilize a different receptor, which in turn may influence viral tropism. We believe that the new data in Figure 3 increases the impact of the manuscript.

**Reviewer #2: None**

**Part III – Minor Issues: Editorial and Data Presentation Modifications**

**Please use this section for editorial suggestions as well as relatively minor modifications of existing data that would enhance clarity.**

**Reviewer #1: (No Response)**

**Reviewer #2: As suggested in my comments above**

Changes made as described above.
