# Supplementary material for: Structure and antigenicity of the divergent human astrovirus VA1 capsid spike
Source: PLoS Pathog. 2024 Feb 28;20(2):e1012028. doi: 10.1371/journal.ppat.1012028 (PMC10950212; doi:10.1371/journal.ppat.1012028)
Supplement: Attachment — Submitted filename: Response to Editor Comments FINAL.docx [file ppat.1012028.s003.docx]

**Response to Editor Comments**

**Editors comment**

Response

We express gratitude to the Editor for the insightful feedback and recommendations, which further enhance the paper's clarity and effectiveness. All line numbers reported below are referring to those in the “Marked up manuscript” file.

**The one remaining issue has to do with the results of the BLI-ISA with rabbit hyperimmune serum. Although they acknowledge in the response to the reviewer that this difference was expected as this serum was raised against the "gastro" variants, the authors omitted to state this in the text. This needs to be fixed.**

We concur and have addressed this by reiterating in the results section that anti-HAstV-VA1^gastro^ polyclonal antibodies were used (line 342), and we added a line in the discussion section “While these results are not entirely surprising given that the antibodies were raised against a gastrointestinal HAstV-VA1 strain, they do…” (lines 405-406).

**Also, in the discussion on the role of trypsin cleavage to release a membrane-lytic peptide that is important for virus entry (paragraph around line 380), something is still not obvious: if HAstV-VA1 undergoes this cleavage intracellularly in the producer cell, the released peptide would presumably lyse the producer cell, but will not be available when it reaches the target cell to be used for entry. It is important to address this issue in the Discussion.**

We agree with the Editor's comment. Upon reflection, we recognize an inaccuracy regarding the term "release" in both the results and discussion sections. The accurate term should be "expose," as demonstrated by Ykema et al. (2013), indicating that trypsin cleavage exposes a membrane-lytic peptide at the carboxy terminus of VP34. Additionally, they further discuss: "VP34 can remain stable in the context of an icosahedral viral capsid when treated with a limited amount of trypsin. Once it encounters a suitable environment, VP34 likely mediates membrane penetration of HAstV8 by inserting its C-terminus into the host membrane." The “suitable environment” may include interaction with the receptor, membrane proximity, acidic pH, additional digestion by host proteases, etc. Considering these observations, the intracellular cleavage of HAstV-VA1 could serve only to prime the virus particle to expose a membrane-lytic peptide, and interactions with the cell receptor or another cue during the virus entry process could induce membrane lysis.

We have expanded the discussion to include these ideas at lines 394-397.

We have also modified line 274 of the results section to be consistent and use the term “exposing.”

**In the Methods section, the statement saying that the multiple amino acid sequence alignment is in Figure 4A needs to be updated, as in the revised version it is Figure 5A (line 428). Also, reading the corresponding paragraph in the Methods, it appears that “all the available neuronal VA1 capsid sequences” amount to six sequences, which is comparable with the five "gastro" sequences used. It is important to state the number of sequences used in the main text (around line 287), as “all the available sequences" is very vague; if there were hundreds of sequences available, it would be normal to find many more differences than in the comparison of 5 sequences of the "gastro" strains.**

We agree and have made the recommended edits as follows:

- In the methods section, we changed Figure 4A to 5A at line 447. We also edited line 451 to state that we used all 6 available neuronal VA1 sequences.
- In the results section, we added clarification as to the number of neuronal HAstV-VA1 capsid sequences publicly available and used in the sequence alignment (lines 291-292 and 296).

**Figure 2: please add rotation symbols to indicate the transformations relating bottom and middle panels.**

We thank the editor for pointing this out and added rotation symbols in Fig 2.

**Line 107: “~35nm T=3 icosahedral capsid” This is the particle diameter, I presume. Please specify.**

We agree and added “diameter” to line 108.

**Lines 116-117: “Notably, while the classical HAstV-1, -2, and -8 spike structures are structurally homologous (RMSD ~1.2 Å)” The word “homologous” (which means that the spike proteins derive from a common ancestor) is misused here, what the authors mean is that the structure of the MLB1 spike has diverged further, but it is still bears homology to the others as the overall fold is retained. Please correct.**

We agree and modified lines 118-120 as “Notably, the classical HAstV-1, -2, and -8 spike structures are structurally similar (RMSD ~1.2 Å), and while the HAstV-MLB1 spike structure retains a similar overall fold, it is more structurally divergent (RMSD ~3.7 Å) and does not have any of the putative receptor-binding sites found on classical HAstV spikes (52,53).”

**Line 213: “sequence homology” should be “sequence similarity”. I urge the authors to read the following commentary on the misuse of the term homology in the literature (https://doi.org/10.1016/0092-8674(87)90322-9)**

We agree and replaced “homology” with “similarity” at line 219.

**Line 216: that classical and HAstV-VA1 “MAY” utilize a different host cell receptor.**

We agree and added “may” into line 221.

---------------------------------------------------------------------------------------------------------------------

Other edits:

1. We made minor edits to Figure 4. In panel A, we reduced the size of the boxes around each letter so they weren’t overlapping with neighboring letters. In panel D, we fixed the linker line between VP34 and VP27 so that there is not a gap.
2. We have changed the authorship order to reflect the additional efforts of Tomás López to generate the new data in Figure 3. All authors agree on this adjustment.
